# Serum inflammatory markers for the screening and diagnosis of periprosthetic joint infection: a systematic review and meta-analysis

**DOI:** 10.5194/jbji-10-363-2025

**Published:** 2025-10-10

**Authors:** Irene K. Sigmund, Matthew J. Dietz, Marta Sabater-Martos, Antony J. R. Palmer, Nicolas Cortés-Penfield, the “Serum Marker Workgroup” for the Unified PJI Definition Taskforce

**Affiliations:** 1 Department of Orthopaedics, Medical University of Vienna, Vienna, Austria; 2 Department of Orthopaedics, Health Sciences Center, WVU School of Medicine, Morgantown, WV, USA; 3 Orthopedic and Traumatology Department, Hospital Clínic de Barcelona, Barcelona, Spain; 4 Nuffield Orthopaedic Centre, Oxford University Hospitals, Oxford, UK; 5 Nuffield Department of Orthopaedics, Rheumatology, and Musculoskeletal Sciences, University of Oxford, Oxford, UK; 6 Division of Infectious Diseases, University of Nebraska Medical Center, Omaha, NE, USA; ➕ A full list of authors appears at the end of the paper

## Abstract

**Aim**: As part of a multi-society effort to derive a unified consensus definition of periprosthetic joint infection (PJI), a systematic review of serum inflammatory marker diagnostic performance for hip, knee, and shoulder PJI was performed. **Methods**: PubMed (MEDLINE) and EMBASE were searched for studies reporting the diagnostic performance of C-reactive protein (CRP), erythrocyte sedimentation rate (ESR), white blood cell count (WBC), fibrinogen, interleukin-6 (IL-6), or D-dimer for PJI. From these, each markers' pooled sensitivity, specificity, positive predictive value (PPV) and negative predictive value (NPV), and area under the summary receiver operating characteristic curve (AUSROC) were calculated using a random-effects model. **Results**: A total of 89 studies reported all diagnostic performance measures for at least one marker. CRP (84 studies, 22 351 patients) demonstrated a pooled sensitivity, specificity, PPV, NPV, and AUSROC of 79.4 % (95 %CI: 78.5–80.3), 77.7 % (77.1–78.3), 67.0 % (63.3–70.7), 86.6 % (84.5–88.7), and 0.872 (SE 0.01), respectively. Corresponding performance estimates for fibrinogen (14 studies, 3433 patients) were 70.9 % (68.3–73.3), 85.9 % (84.3–87.3), 77.2 % (71.8–82.6), 82.1 % (77.1–87.2), and 0.889 (0.02), respectively, and those for IL-6 (20 studies, 2318 patients) were 76.3 % (73.4–79.0), 85.8 % (83.8–87.6), 74.5 % (69.0–80.0), 86.0 % (80.6–91.3), and 0.900 (0.01), respectively. ESR, D-dimer, and WBC did not offer greater predictive values than these markers. **Conclusion**: Although serum CRP, fibrinogen, and IL-6 demonstrated the best performance among all analysed parameters, their diagnostic accuracy remains insufficient to reliably confirm or exclude PJI. Elevated serum markers should be re-evaluated as a diagnostic criterion in future PJI definitions. **Level of evidence**: The level of evidence was Level III.

## 1 Introduction

Serum inflammatory markers are widely used to aid the diagnosis of periprosthetic joint infection (PJI). They are easy to obtain, available globally, cheap, and deliver timely results. However, the accuracies of serum inflammatory markers for PJI are limited by their poor differentiation between septic and aseptic failure after total joint arthroplasty, particularly among patients with underlying inflammatory disorders.

Which (or whether) serum inflammatory markers should be used for PJI diagnosis remains unclear. The 2019 guideline of the American Association of Orthopaedic Surgeons (AAOS) recommends serum C-reactive protein (CRP), erythrocyte sedimentation rate (ESR), and interleukin 6 (IL-6) in the preoperative evaluation of PJI (Aaos, 2019). The 2018 revised International Consensus Meeting (ICM) definition (Shohat et al., 2019) includes elevated CRP, ESR, and D-dimer as minor diagnostic criteria. The Musculoskeletal Infection Society (MSIS) definitions from 2011 and 2013 (Parvizi et al., 2011; Parvizi and Gehrke, 2014) also incorporate CRP and ESR as minor criteria. In contrast, the 2021 definition proposed by the European Bone and Joint Infection Society (EBJIS) includes only serum CRP as a serum biomarker (McNally et al., 2021). Notably, the Infectious Disease Society of America (IDSA) guideline does not include any serum markers in its diagnostic criteria for PJI infection definition (Osmon et al., 2013).

The EBJIS, ICM, MSIS, IDSA, and the European Society of Clinical Microbiology and Infectious Diseases Study Group for Implant-Associated Infections (ESCMID-ESGIAI) have recently convened a joint taskforce to develop a unified consensus definition of PJI. As part of that effort, a systematic review and meta-analysis was conducted to assess the performance of sufficiently studied serum inflammatory markers for the preoperative diagnosis of PJI.

## 2 Methods

On behalf of the “Serum Marker Workgroup” of the Unified PJI Definition Taskforce, a systematic review and meta-analysis of the value of serum inflammatory markers for the diagnosis of hip, knee, and/or shoulder PJI was performed based on the Preferred Reporting Items for Systematic reviews and Meta-Analyses (PRISMA) guidelines (Page et al., 2021). The pooled accuracy estimates of each sufficiently studied serum inflammatory parameter were calculated, and markers were intercompared to find those most accurate. In addition, the best thresholds to differentiate between septic and aseptic arthroplasty failure for each serum parameter were evaluated. Given the great between-study heterogeneity in reference standards for infection, specific patient populations studied, and diagnostic thresholds for positivity in this literature base, only serum inflammatory markers evaluated in at least 10 studies on their diagnostic value in periprosthetic joint infection (PJI) were considered *sufficiently studied* and included in this systematic review and meta-analysis.

### 2.1 Search strategy and screening

Search queries for PubMed (MEDLINE) and EMBASE were developed to capture all clinical studies reporting diagnostic performance measures of one or more serum inflammatory markers for PJI (see Table S1 in the Supplement for full queries). The serum markers included in the search query were as follows: ESR; CRP; IL-6; D-dimer; fibrinogen; white blood cell count (WBC); procalcitonin; platelet volume; and the various ratios involving serum neutrophils, lymphocytes, monocytes, and/or immune globulins. The literature search was conducted on 1 February 2024. All retrieved titles and abstracts were divided into three equal sets, with each set screened once for eligibility by one of three authors (Irene K. Sigmund, Matthew J. Dietz, and Nicolas Cortés-Penfield). The resulting eligible studies were then again divided into three sets, and each author reviewed the full texts of their assigned set once, applying the predefined inclusion and exclusion criteria and documenting reasons for exclusion. Additionally, relevant systematic reviews identified in the search were screened to identify any further eligible studies not captured initially.

### 2.2 Inclusion and exclusion criteria

English-language studies assessing the diagnostic value of at least one serum inflammatory marker in diagnosing PJI after a total hip, knee, or shoulder arthroplasty were considered for inclusion. Further inclusion criteria were that (1) the study clearly stated the reference standard for PJI diagnosis and (2) it reported the sensitivity, specificity, positive predictive value (PPV), and negative predictive value (NPV) for at least one serum inflammatory marker. Studies including infections other than PJI, case reports, and non-human studies were excluded.

### 2.3 Data extraction

Relevant study characteristics and diagnostic accuracy data (i.e. sensitivity, specificity, PPV, and NPV) from each eligible study were extracted by three authors (Irene K. Sigmund, Matthew J. Dietz, and Nicolas Cortés-Penfield) using a standardized form. Relevant study characteristics included the PJI reference standard; number of patients with an affected knee, hip, or shoulder; number of patients with PJI and aseptic failure; and the cutoff value for an abnormal test result used as well as the origin of that cutoff (i.e. prespecified vs. derived post hoc from the Youden index).

### 2.4 Quality assessment

The Quality Assessment of Diagnostic Accuracy Studies (QUADAS-2) tool was used to assess the risk of bias and applicability to this meta-analysis across four domains, including patient selection, index test, reference standard, and enrolment flow of patients/timing of the index and reference tests (Whiting et al., 2011). Risk of bias and applicability were rated as “low”, “high”, or “unclear” based on the provided study information, and the quality of each included study was then graded from A (high) to D (very low). Quality assessment was performed by the author assigned to each specific article during the full-text review and data extraction. Each study was reviewed by a single author. In cases of uncertainty or ambiguity, a second author re-reviewed the article, and any discrepancies were resolved through discussion to reach a consensus.

### 2.5 Statistical analysis

MetaDiSc 1.4^®^ and RStudio 4.4.1^®^ were used for quantitative analyses of each serum inflammatory biomarker. The number of true positives, false positives, true negatives, and false negatives for each serum inflammatory parameter were calculated using the number of PJI and aseptic cases and the given sensitivities and specificities in each study. The pooled estimates for sensitivity, specificity, positive predictive value (PPV) and negative predictive value (NPV), positive likelihood ratio (LR + ) and negative likelihood ratio (LR - ), and the area under the summary receiver operating characteristic curve (AUSROC) were calculated with their 95 % confidence intervals (CIs). The diagnostic odds ratio (DOR) was evaluated to measure the effectiveness of diagnostic testing. Statistical heterogeneity for each accuracy measure was determined by Higgins $I^{2}$ statistic ( $I^{2}$ values > 50 % reflect substantial heterogeneity and should be interpreted with caution; Higgins and Thompson, 2002). Scatterplots comparing Youden indices with cutoffs were used to determine the optimal cutoff for each parameter using jamovi (version 2.2.5). For parameters providing the required data, the optimal cutoff for only hips or only knees was calculated. Due to the limited number of data for shoulders, no detailed analyses could be performed.

## 3 Results

### 3.1 Study identification and inclusion

**Figure 1:**
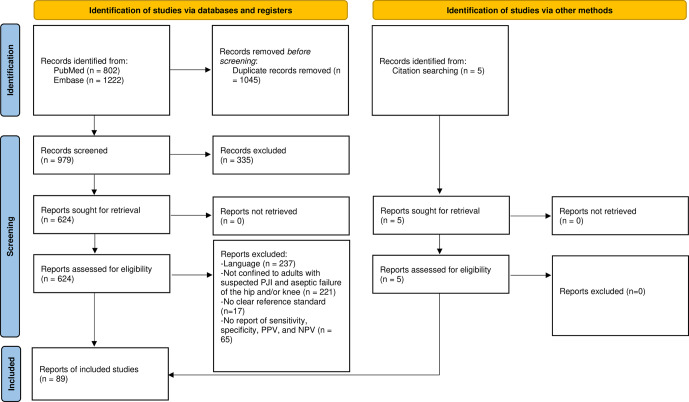
PRISMA flow diagram of the literature screening process.

The initial search yielded 802 results from PubMed and 1222 results from EMBASE. After automated reference extraction with the removal of missing and duplicate references using EndNote and the elimination of irrelevant references via title screening, 624 references underwent abstract screening. After applying the inclusion and exclusion criteria and screening the references of prior relevant systematic reviews identified in the search, we included a total 89 studies in this meta-analysis (Fig. 1).

Of the serum inflammatory markers included in the search query, only CRP, ESR, WBC, fibrinogen, IL-6, and D-dimer met the criteria for inclusion in our meta-analysis. Serum procalcitonin, platelet volume, and ratios of specific cell lines and/or immune globulins all had ≤ 10 included studies reporting their accuracy in PJI diagnosis and were not considered further.

In total, 84 studies (including 22 351 patients) evaluated the diagnostic potential of serum CRP (Abou El-Khier et al., 2013; Ackmann et al., 2020; Ahmadi et al., 2018; Alijanipour et al., 2013; Austin et al., 2008; Bare et al., 2006; Berger et al., 2017; Bernard et al., 2004; Bin et al., 2020; Bottner et al., 2007; Buttaro et al., 2010; Cao et al., 2020; Chen et al., 2021; Chisari et al., 2021; Cipriano et al., 2012; Claassen et al., 2016; Deirmengian et al., 2010; Deirmengian et al., 2021; Della Valle et al., 2007; Denyer et al., 2023; Di Cesare et al., 2005; Ding et al., 2019; Dong et al., 2024; Elgeidi et al., 2014; Erdemli et al., 2018; Ettinger et al., 2015; Fernández-Sampedro et al., 2017; Fernandez-Sampedro et al., 2022; Fink et al., 2008; Fink et al., 2013; Fink et al., 2018; Fink et al., 2020; Fu et al., 2019; Ghanem et al., 2009; Greidanus et al., 2007; Grzelecki et al., 2021; Hu et al., 2020; Huang et al., 2022; Itasaka et al., 2001; Klemt et al., 2023; Klim et al., 2024; Kuo et al., 2018; Kuo et al., 2022; Levent et al., 2021; Li et al., 2019; Liu et al., 2014; Liu et al., 2022; Maimaiti et al., 2022; Majors and Jagadale, 2019; Muñoz-Mahamud et al., 2022; Nilsdotter-Augustinsson et al., 2007; Parvizi et al., 2012; Paziuk et al., 2020; Piper et al., 2010; Qin et al., 2020a; Qin et al., 2020b; Schinsky et al., 2008; Shah et al., 2016; Shahi et al., 2017; Shang et al., 2022; Shi et al., 2023; Sigmund et al., 2021; Tetreault et al., 2014; Tirumala et al., 2021; Tohtz et al., 2010; Villacis et al., 2014; Wang et al., 2020, 2021, 2023a, b; Worthington et al., 2010; Wouthuyzen-Bakker et al., 2018; Wu et al., 2014, 2020, 2023; Xu et al., 2019, 2020, 2021, 2022; Yang et al., 2021; Ye et al., 2021; Yin et al., 2021; Yu et al., 2020, 2021), 67 studies (including 19660 patients) evaluated the diagnostic potential of ESR (Abou El-Khier et al., 2013; Ahmadi et al., 2018; Alijanipour et al., 2013; Austin et al., 2008; Bare et al., 2006; Berger et al., 2017; Bernard et al., 2004; Bottner et al., 2007; Buttaro et al., 2010; Chen et al., 2021; Chisari et al., 2021; Cipriano et al., 2012; Deirmengian et al., 2010; Deirmengian et al., 2021; Della Valle et al., 2007; Denyer et al., 2023; Di Cesare et al., 2005; Ding et al., 2019; Dong et al., 2024; Elgeidi et al., 2014; Fernandez-Sampedro et al., 2022; Fu et al., 2019; Ghanem et al., 2009; Greidanus et al., 2007; Grzelecki et al., 2021; Hu et al., 2020; Huang et al., 2022; Itasaka et al., 2001; Klemt et al., 2023; Kuo et al., 2018; Kuo et al., 2022; Li et al., 2019; Liu et al., 2014, 2022; Maimaiti et al., 2022; Majors and Jagadale, 2019; Muñoz-Mahamud et al., 2022; Nilsdotter-Augustinsson et al., 2007; Paziuk et al., 2020; Piper et al., 2010; Qin et al., 2020a, b; Schinsky et al., 2008; Shah et al., 2016; Shahi et al., 2017; Shang et al., 2022; Shi et al., 2023; Tirumala et al., 2021; Tohtz et al., 2010; Villacis et al., 2014; Wang et al., 2020, 2021, 2023a, 2023b; Worthington et al., 2010; Wouthuyzen-Bakker et al., 2018; Wu et al., 2014, 2020, 2023; Xu et al., 2019, 2020, 2021, 2022; Yang et al., 2021; Ye et al., 2021; Yu et al., 2020; Chen et al., 2022), 14 studies (including 3550 patients) evaluated the diagnostic potential of WBC (Abou El-Khier et al., 2013; Bottner et al., 2007; Di Cesare et al., 2005; Elgeidi et al., 2014; Itasaka et al., 2001; Klim et al., 2024; Li et al., 2019; Maimaiti et al., 2022; Sigmund et al., 2021; Tohtz et al., 2010; Toossi et al., 2012; Villacis et al., 2014; Yang et al., 2021; Yu et al., 2020), 14 studies (including 3433 patients) evaluated the diagnostic potential of fibrinogen (Bin et al., 2020; Chen et al., 2021, 2022; Chisari et al., 2021; Dong et al., 2024; Li et al., 2019; Maimaiti et al., 2022; Sigmund et al., 2021; Wang et al., 2020; Wu et al., 2020, 2023; Xu et al., 2020, 2022; Yang et al., 2021), 20 studies (including 2318 patients) evaluated the diagnostic potential of IL-6 (Abou El-Khier et al., 2013; Ackmann et al., 2020; Bottner et al., 2007; Buttaro et al., 2010; Di Cesare et al., 2005; Elgeidi et al., 2014; Erdemli et al., 2018; Ettinger et al., 2015; Gallo et al., 2018; Gollwitzer et al., 2013; Majors and Jagadale, 2019; Qin et al., 2020b; Villacis et al., 2014; Worthington et al., 2010; Xu et al., 2019, 2021, 2022; Yin et al., 2021; Yu et al., 2020, 2021), and 21 studies (including 3177 patients) evaluated the diagnostic potential of D-dimer (Ackmann et al., 2020; Chen et al., 2021; Chisari et al., 2021; Dong et al., 2024; Fernandez-Sampedro et al., 2022; Fu et al., 2019; Grzelecki et al., 2021; Hu et al., 2020; Kuo et al., 2022; Li et al., 2019; Liu et al., 2022; Maimaiti et al., 2022; Muñoz-Mahamud et al., 2022; Shahi et al., 2017; Wu et al., 2020; Xu et al., 2019, 2021; Chen et al., 2022; Pannu et al., 2020; Qin et al., 2020a; Wang et al., 2020). Full characteristics of all included studies are provided in Tables S2–S7.

### 3.2 Quality assessment

Of the 89 included studies, 24 were graded as moderate (B), 48 as low (C), and 17 as very low (D) using the QUADAS-2 risk of bias assessment. No study was graded as high quality (A). Risk of bias and applicability concerns are shown in Table 1. In the majority of studies (84 % n= 75/89), the overall risk of bias regarding the reference standard was high, with significant concerns about the applicability of the reference standard in 67 % of studies ( n= 60/89). Incorporation bias (i.e. use of PJI diagnostic criteria that include the inflammatory markers being studied as the reference standard) was a consistent issue with these studies and may have inflated the apparent accuracy of ESR, CRP, and/or D-dimer relative to other markers.

**Table 1:** Risk of bias (RoB) of all included studies based on the QUADAS-2 classification system.

Study	RoB in	Applicability in	RoB in	Applicability	RoB in	Applicability	RoB in	Conflict of interest,	Grade
(year)	patient selection	patient selection	index test	in index test	reference standard	in reference standard	patient flow	financial conflict	classification
Abou El-Khier (2013)	High risk	High risk	Low risk	Low risk	High risk	High risk	Low risk	No	D
Ackmann et al. (2020)	High risk	Low risk	Low risk	Low risk	High risk	High risk	Low risk	No	C
Ahmadi et al. (2018)	Low risk	Low risk	Low risk	Low risk	High risk	High risk	Low risk	No	C
Alijanipour et al. (2013)	High risk	Low risk	Unclear	Low risk	Low risk	Low risk	Low risk	No	B
Austin et al. (2008)	Low risk	Low risk	Low risk	Low risk	High risk	Low risk	Low risk	No	B
Bare et al. (2006)	Low risk	Low risk	Low risk	Low risk	High risk	High risk	Low risk	No	C
Berger et al. (2017)	Low risk	Low risk	Low risk	Low risk	High risk	Unclear	Low risk	No	B
Bernard et al. (2004)	Low risk	Low risk	Low risk	Low risk	High risk	Low risk	Low risk	No	B
Bin et al. (2020)	Low risk	High risk	High risk	High risk	High risk	Low risk	Low risk	No	C
Bottner et al. (2007)	High risk	Low risk	Low risk	Unclear	High risk	Unclear	Low risk	No	C
Buttaro et al. (2010)	High risk	Low risk	Low risk	Low risk	High risk	High risk	Low risk	No	D
Cao et al. (2020)	Low risk	Low risk	High risk	Low risk	High risk	Low risk	Low risk	No	C
Chen et al. (2021)	High risk	Unclear	High risk	High risk	High risk	High risk	Low risk	No	D
Chen et al. (2022)	High risk	Low risk	High risk	Low risk	Low risk	High risk	Low risk	No	C
Chisari et al. (2021)	Low risk	Unclear	Unclear	Low risk	High risk	Low risk	Low risk	No	B
Cipriano et al. (2012)	Low risk	Unclear	High risk	Unclear	High risk	Unclear	Low risk	No	C
Claassen et al. (2016)	Low risk	Unclear	Low risk	High risk	High risk	High risk	Low risk	No	D
Deirmengian et al. (2010)	Low risk	Low risk	Low risk	Low risk	High risk	High risk	Low risk	No	B
Deirmengian et al. (2021)	Low risk	Low risk	Low risk	Low risk	High risk	Low risk	Low risk	No	B
Della Valle et al. (2007)	Low risk	Low risk	Low risk	Low risk	High risk	High risk	Low risk	No	C
Denyer et al. (2023)	Low risk	Unclear	Low risk	Low risk	High risk	Low risk	Low risk	No	B
Di Cesare et al. (2005)	High risk	High risk	Unclear	Low risk	High risk	High risk	Low risk	No	D
Ding et al. (2019)	Low risk	Low risk	Low risk	Low risk	High risk	Low risk	Low risk	No	B
Dong et al. (2024)	Unclear	Unclear	High risk	Unclear	High risk	Unclear	Low risk	No	D
Elgeidi et al. (2014)	High risk	High risk	High risk	Low risk	High risk	High risk	Low risk	Unclear	D
Erdemli et al. (2018)	Low risk	Low risk	High risk	High risk	High risk	Low risk	Low risk	No	C
Ettinger et al. (2015)	High risk	High risk	Unclear	Low risk	High risk	High risk	Low risk	No	C
Fernández-Sampedro et al. (2017)	Low risk	Low risk	Low risk	Low risk	High risk	High risk	Low risk	No	C
Fernandez-Sampedro et al. (2022)	Low risk	Low risk	High risk	Low risk	High risk	High risk	Low risk	No	D
Fink et al. (2008)	Low risk	Low risk	Low risk	Low risk	High risk	High risk	Low risk	No	B
Fink et al. (2013)	Low risk	Low risk	Low risk	Low risk	High risk	High risk	Low risk	No	B
Fink et al. (2018)	Low risk	Low risk	Low risk	Low risk	High risk	High risk	Low risk	No	B
Fink et al. (2020)	High risk	Low risk	Low risk	Low risk	High risk	High risk	Low risk	No	C
Fu et al. (2019)	High risk	Low risk	Low risk	Unclear	High risk	High risk	Low risk	No	C
Gallo et al. (2018)	High risk	Low risk	High risk	Low risk	High risk	High risk	Low risk	No	D
Ghanem et al. (2009)	Low risk	Low risk	Unclear	Low risk	High risk	High risk	Low risk	No	B
Gollwitzer et al. (2013)	High risk	Unclear	High risk	Low risk	High risk	High risk	Low risk	No	D
Greidanus et al. (2007)	Low risk	Low risk	High risk	High risk	High risk	High risk	Low risk	No	C
Grzelecki et al. (2021)	High risk	Unclear	Unclear	Unclear	High risk	Low risk	Low risk	No	C
Hu et al. (2020)	Low risk	Low risk	High risk	Low risk	High risk	Low risk	Low risk	No	C
Huang et al. (2022)	Low risk	Low risk	High risk	Low risk	High risk	Low risk	Low risk	No	C
Itasaka et al. (2001)	Low risk	Low risk	Low risk	Low risk	High risk	High risk	Low risk	No	B
Klemt et al. (2023)	High risk	Low risk	Low risk	Unclear	High risk	High risk	Low risk	No	C
Klim et al. (2024)	Unclear	Unclear	Low risk	High risk	High risk	High risk	Low risk	No	C
Kuo et al. (2018)	High risk	High risk	Low risk	Low risk	Low risk	Low risk	Low risk	No	B
Kuo et al. (2022)	Low risk	Unclear	Low risk	Unclear	High risk	High risk	High risk	No	C
Levent et al. (2021)	High risk	High risk	High risk	High risk	Low risk	Low risk	Unclear	No	C
Li et al. (2019)	Low risk	Low risk	Low risk	Low risk	High risk	High risk	Low risk	No	B
Liu et al. (2014)	High risk	High risk	High risk	High risk	Low risk	Low risk	Unclear	No	C
Liu et al. (2022)	High risk	High risk	Low risk	Low risk	Low risk	Low risk	Unclear	No	B
Maimaiti et al. (2022)	High risk	Low risk	Low risk	Low risk	High risk	High risk	Low risk	No	C
Majors et al. (2019)	High risk	High risk	High risk	Low risk	High risk	High risk	Low risk	No	C
Muñoz-Mahamud et al. (2022)	High risk	High risk	Unclear	Low risk	High risk	High risk	Low risk	Unclear	C
Nilsdotter-Augustinsson et al. (2007)	High risk	High risk	High risk	High risk	High risk	High risk	Unclear	No	C
Pannu et al. (2020)	Unclear	Low risk	Low risk	Low risk	Low risk	Low risk	Low risk	Unclear	B
Parvizi et al. (2012)	High risk	High risk	Low risk	Low risk	Low risk	Low risk	Unclear	No	B
Paziuk et al. (2020)	High risk	Low risk	High risk	Low risk	High risk	High risk	Low risk	Unclear	C
Piper et al. (2010)	High risk	High risk	Low risk	Low risk	Low risk	Low risk	Unclear	No	B
Qin et al. (2020a)	High risk	Low risk	Low risk	Unclear	High risk	High risk	Low risk	No	B
Qin et al. (2020b)	High risk	High risk	Low risk	Low risk	High risk	High risk	Low risk	No	C
Schinsky et al. (2008)	High risk	High risk	Low risk	High risk	High risk	High risk	Low risk	No	C
Shah et al. (2016)	High risk	Low risk	High risk	Low risk	Low risk	Low risk	High risk	No	C
Shahi et al. (2017)	High risk	Low risk	Low risk	Unclear	High risk	High risk	Low risk	No	C
Shang et al. (2022)	High risk	Low risk	High risk	Low risk	Low risk	Low risk	Low risk	No	C
Shi et al. (2023)	High risk	High risk	High risk	Low risk	Low risk	Low risk	Low risk	No	C
Sigmund et al. (2021)	Low risk	Low risk	High risk	Low risk	Low risk	Low risk	Low risk	No	B
Tetreault et al. (2014)	Low risk	Low risk	High risk	Low risk	High risk	High risk	Low risk	Unclear	C
Tirumala et al. (2021)	High risk	High risk	High risk	High risk	High risk	High risk	High risk	Unclear	D
Tohtz et al. (2010)	Low risk	Low risk	Low risk	Unclear	High risk	High risk	Low risk	No	D
Toossi et al. (2012)	Low risk	Low risk	High risk	Unclear	High risk	High risk	Low risk	Unclear	C
Villacis et al. (2014)	High risk	High risk	High risk	High risk	High risk	High risk	High risk	No	D
Wang et al. (2020)	High risk	Low risk	High risk	Low risk	High risk	High risk	High risk	No	C
Wang et al. (2021)	High risk	High risk	Low risk	High risk	High risk	High risk	Low risk	No	C
Wang et al. (2023a)	High risk	Low risk	High risk	Low risk	High risk	High risk	Low risk	No	C
Wang et al. (2023b)	High risk	Low risk	High risk	High risk	High risk	High risk	Low risk	No	C
Worthington et al. (2010)	High risk	Low risk	High risk	High risk	Unclear	High risk	Low risk	Unclear	D
Wouthuyzen-Bakker et al. (2018)	Low risk	Low risk	High risk	High risk	High risk	High risk	Low risk	No	D
Wu et al. (2014)	High risk	High risk	Low risk	High risk	High risk	High risk	Low risk	No	C
Wu et al. (2020)	High risk	Low risk	Low risk	Unclear	High risk	High risk	High risk	No	C
Wu et al. (2023)	High risk	Low risk	High risk	Unclear	High risk	High risk	Low risk	No	C
Xu et al. (2019)	Low risk	Low risk	Low risk	Unclear	High risk	High risk	Low risk	No	B
Xu et al. (2020)	High risk	Low risk	High risk	Unclear	High risk	Low risk	Low risk	Unclear	C
Xu et al. (2021)	High risk	Low risk	High risk	Low risk	High risk	High risk	Low risk	No	C
Xu et al. (2022)	Low risk	Low risk	High risk	Unclear	High risk	High risk	Low risk	No	C
Yang et al. (2021)	High risk	Low risk	High risk	Unclear	High risk	High risk	Low risk	No	C
Ye et al. (2021)	High risk	Low risk	High risk	Unclear	High risk	High risk	Low risk	No	C
Yin et al. (2021)	Low risk	Low risk	Low risk	Low risk	High risk	High risk	Low risk	Yes	D
Yu et al. (2020)	High risk	Unclear	Low risk	High risk	High risk	High risk	Low risk	No	D
Yu et al. (2021)	Low risk	Low risk	Low risk	Unclear	High risk	High risk	Low risk	No	B

### 3.3 Diagnostic potential of serum inflammatory markers

**Figure 2:**
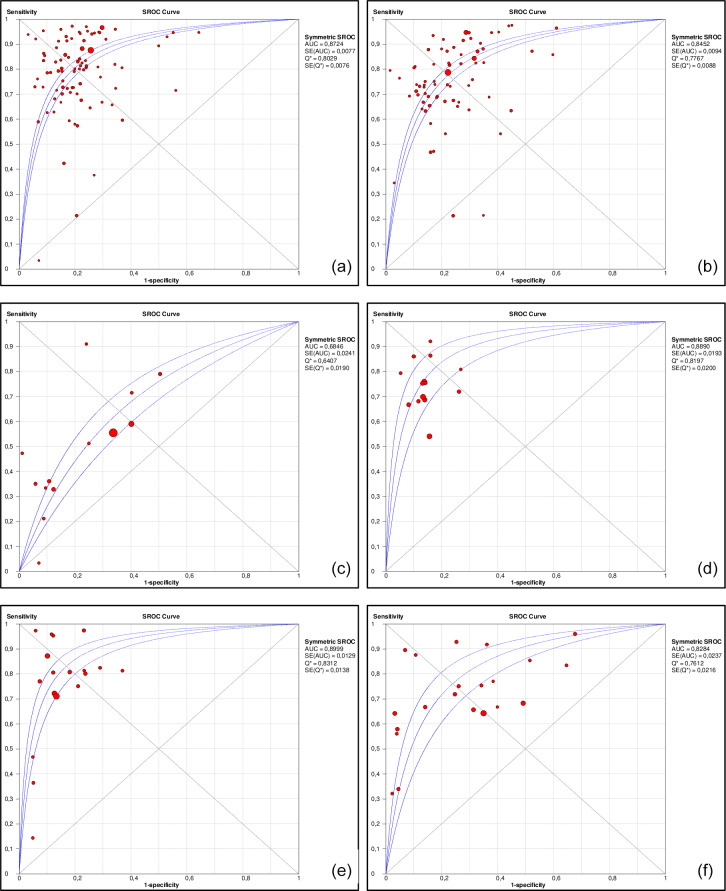
Area under the summary receiver operating characteristic curves (AUSROCs) for C-reactive protein (CRP, **a**), erythrocyte sedimentation rate (ESR, **b**), white blood cell count (WBC, **c**), fibrinogen **(d)**, interleukin-6 **(e)**, and D-dimer **(f)**.

The detailed performance estimates of serum C-reactive protein (CRP), erythrocyte sedimentation rate (ESR), white blood cell count (WBC), fibrinogen, interleukin-6, and D-dimer are illustrated in Table 2. Summarized receiver operating curves of all serum parameters are illustrated in Fig. 2. The highest Youden index was observed at cutoffs of 10.7 mg L^−1^ (95 %CI: 9.8–11.6) for CRP, 30.6 mm h^−1^ (29.2–32.0) for ESR, 9.13 G L^−1^ (8.3–10.0) for WBC, 3.8 g L^−1^ (3.7–4.0) for fibrinogen, 9.8 pg mL^−1^ (7.8–11.8) for IL-6, and 1.1 mg L^−1^ (0.8–1.4) for D-dimer. However, all parameters demonstrated a low Spearman correlation ( < 0.6) without a significant threshold effect. Meta-regression revealed that the diagnostic threshold used for ESR and CRP is not associated with diagnostic performance.

**Table 2:** Pooled sensitivity, specificity, positive predictive value (PPV) and negative predictive value (NPV), positive likelihood ratio (LR + ) and negative likelihood ratio (LR - ), diagnostic odds ratio (DOR), and area under the summary receiver operating characteristic curve (AUSROC) for serum C-reactive protein (CRP), erythrocyte sedimentation rate (ESR), white blood cell count (WBC), fibrinogen, interleukin-6, and D-dimer.

Serum	Advocated	No. of	Total no.	Sensitivity	$I_{\mathrm{Sen}}^{2}$	Specificity	$I_{\mathrm{Spec}}^{2}$	PPV	$I_{\mathrm{PPV}}^{2}$	NPV	$I_{\mathrm{NPV}}^{2}$	LR +	$I_{\mathrm{LR}+}^{2}$	LR-	$I_{\mathrm{LR}-}^{2}$	DOR	$I_{\mathrm{DOR}}^{2}$	AUSROC (SE)
markers	cutoffs	included studies	of patients	(95%CI)	(%)	(95%CI)	(%)	(95%CI)	(%)	(95%CI)	(%)	(95%CI)	(%)	(95%CI)	(%)	(95%CI)	(%)	
CRP	> 10 mg L^−1^	84	22 351	79.4 %	90.7	77.7 %	84.9	67.0 %	98.0	86.6 %	100	3.73	82.7	0.24	94.7	17.01	82.8	0.872
				(78.5–80.3)		(77.1–78.3)		(63.3–70.7)		(84.5–88.7)		(3.43–4.06)		(0.20–0.29)		(13.81–20.95)		(0.01)
ESR	> 30 mm h^−1^	67	19 660	75.5 %	90.2	75.5 %	89.7	62.4 %	100	85.0 %	100	3.19	86.1	0.30	92.6	11.98	81.7	0.845
				(74.4–76.6)		(74.8–76.2)		(57.8–67.0)		(82.5–87.6)		(2.90–3.51)		(0.25–0.35)		(9.70–14.80)		(0.01)
WBC	Not advocated	14	3550	52.4 %	87.0	69.5 %	93.2	54.0 %	100	76.0 %	97.1	2.14	62.4	0.70	75.0	3.61	47.4	0.685
				(49.6–55.2)		(67.6–71.4)		(40.5–67.5)		(67.3–84.7)		(1.74–2.63)		(0.60–0.81)		(2.58–5.04)		(0.02)
Fibrinogen	> 4 g L^−1^	14	3433	70.9 %	75.2	85.9 %	51.4	77.2 %	93.5	82.1 %	94.4	5.03	57.2	0.31	75.2	17.32	65.9	0.889
				(68.3–73.3)		(84.3–87.3)		(71.8–82.6)		(77.1–87.2)		(4.18–6.05)		(0.26–0.38)		(12.34–24.31)		(0.02)
IL-6	> 10 pg mL^−1^	20	2318	76.3 %	80.7	85.8 %	60.6	74.5 %	90.0	86.0 %	100	5.05	50.8	0.28	87.6	21.5	42.9	0.900
				(73.4–79.0)		(83.8–87.6)		(69.0–80.0)		(80.6–91.3)		(4.09–6.22)		(0.20–0.40)		(14.89–31.05)		(0.01)
D-dimer	> 1 mg L^−1^	21	3177	69.9 %	86.5	72.9 %	94.2	68.9 %	98.7	78.2 %	96.4	3.13	90.4	0.39	80.4	10.60	82.5	0.828
				(67.2–72.5)		(70.9–74.8)		(60.6–77.1)		(72.0–84.4)		(2.36–4.14)		(0.32–0.48)		(6.50–17.30)		(0.02)

Other terms used in the table are as follows: CI – confidence interval; $I^{2}$ – I square, inconsistency.

Among the analysed serum inflammatory markers, CRP, fibrinogen, and IL-6 demonstrated the best performance for diagnosing PJI. Serum CRP offered superior sensitivity compared to fibrinogen (79 % [95 %CI 78.5–80.3] vs. 71 % [95 %CI 68.3–73.3]) but inferior specificity (77.7 % [77.1–78.3] vs. 85.9 % [84.3–87.3]). IL-6 also offered superior sensitivity compared to fibrinogen (76.3 % [73.4–79.0] vs. 69.9 % [67.2–72.5]) but with comparable specificity (85.8 % [83.8–87.6] vs. 85.9 % [84.3–87.3]). ESR sensitivity was inferior to CRP, comparable to IL-6, and slightly superior to fibrinogen, but its specificity was inferior to all three, making it no better as a rule-out test and less useful as a rule-in test vs. the aforementioned comparators. As for the other inflammatory markers, D-dimer demonstrated sensitivity similar to fibrinogen and inferior to alternatives, with specificity similar to ESR and inferior to alternatives, while serum WBC demonstrated little to no diagnostic utility. $I^{2}$ values for nearly every diagnostic parameter for every marker exceeded 50 %, suggesting substantial between-study heterogeneity.

Only CRP and ESR had sufficient studies reporting their diagnostic utility in hip vs. knee PJI to facilitate stratified analyses. CRP appeared modestly less sensitive for knee vs. hip PJI (75.9 % [95 %CI 73.0–78.7] vs. 83.1 % [95 %CI: 79.8–86.0]) but similarly specific, with a comparable overall AUSROC (0.848 [SE 0.01] vs. 0.850 [SE 0.03]) and optimal diagnostic thresholds (9.1 mg L^−1^ [7.1–11.2] vs. 11.1 mg L^−1^ [8.9–13.3]). ESR appeared similar in both sensitivity (79.9 % [75.9–83.4] vs. 84.4 % [82.1–87.2]) and specificity (69.9 % [67.1–72.7] vs. 71.6 % [68.9–74.2]) for hip vs. knee PJI, with comparable optimal diagnostic thresholds (30 mm h^−1^ [30–30] vs. 28.8 mm h^−1^ [26.9–30.8]). These findings need to be interpreted with caution due to the small number of studies included and the heterogeneity of inclusion and exclusion criteria of the analysed studies. No marker had sufficient studies reporting accuracy in shoulder PJI for a stratified analysis.

Reporting of PJI timing and/or chronicity (i.e. early postoperative vs. acute hematogenous vs. chronic PJI) was limited and inconsistent across the identified studies; we judged the data insufficient to derive optimal diagnostic thresholds of any inflammatory marker for acute PJI. Similarly, data were insufficient to perform sub-analyses of markers' relative diagnostic utilities in specific patient populations (e.g. those with underlying inflammatory disorders).

## 4 Discussion

Among the analysed serum inflammatory markers, CRP, fibrinogen, and IL-6 demonstrated the best performance for diagnosing PJI and were largely equivalent with respect to performance. Serum CRP offered a slightly superior sensitivity (79 %) compared to fibrinogen (71 %) and IL-6 (76 %) but inferior specificity (CRP: 78 %; fibrinogen: 86 %; IL-6: 86 %), leading to a lower PPV and similar NPV. Relative to CRP and fibrinogen, IL-6 is less widely available as an in-house test and may be more expensive in comparison to the other serum parameters.

While ESR has a long history of use in PJI diagnosis and was incorporated into previous PJI diagnostic criteria, its relative lack of specificity translated into an NPV that was no better than that for CRP, fibrinogen, or IL-6 and a similar or worse PPV. Serum WBC was not valuable as a diagnostic tool, nor was D-dimer, which is incorporated into the 2018 ICM criteria for PJI and was inferior with respect to sensitivity and/or specificity to all other serum biomarkers studied. We, therefore, recommend future definitions do not use WBC, ESR, or D-dimer as criteria.

Although a cutoff of 10.7 mg L^−1^ for CRP demonstrated the best Youden index in our scatterplot analysis, it was not substantially superior to the established cutoff of 10 mg L^−1^ (despite post hoc derivations of optimal cutoffs likely overestimating their true utility). In meta-regression analysis, the diagnostic threshold used for ESR and CRP in each study was not associated with diagnostic performance. For fibrinogen and IL-6, no consensus cutoff value was consistently used among the included studies, and optimal Youden indices were observed with cutoffs of 3.8 g L^−1^ and 9.8 pg mL^−1^, respectively. Given that most studies used a diagnostic threshold of 10 mg L^−1^ for CRP and that alternative threshold did not improve accuracy, we recommend the adoption of this threshold. Based on our analysis, thresholds of > 10 mg L^−1^ for CRP, > 4 g L^−1^ for fibrinogen, and > 10 pg mL^−1^ for IL-6 can be proposed.

Data on the performance of the serum inflammatory parameters at one site (hip, knee, or shoulder) were only available for CRP and ESR. Compared to knees, hips offered a substantially superior sensitivity (83.1 % vs. 75.9 %) and similar specificity (76.6 % vs. 76.8 %) of CRP. Hence, it seems that CRP performs better in hips. Interestingly, the opposite was observed when analysing ESR; knees demonstrated a slightly superior sensitivity (84.4 % vs. 79.9 %) and equivalent specificity (71.6 % vs. 69.9 %). However, these findings need to be interpreted with caution due to the small number of studies included and the heterogeneity in the inclusion and exclusion criteria of the analysed studies. Based on these findings, no definitive conclusion regarding the affected joint can be drawn.

However, due to their overall insufficient diagnostic accuracy, all of these serum inflammatory markers cannot be considered reliable stand-alone tests for PJI. Given these limitations and the increasing evidence supporting the higher sensitivity and specificity of synovial vs. serum inflammatory markers for PJI, it is time to reconsider the inclusion of serum markers in future definitions of periprosthetic joint infection.

Due to the low sensitivities, a negative result of these markers cannot rule out PJI. The high number of false negatives can be explained by the inadequate immune response in PJIs caused by low-virulence microorganisms (i.e.; coagulase-negative staphylococci and *Cutibacterium* spp., Akgün et al., 2018) and in PJIs with draining sinus tracts. In addition, patients with an impaired immune system or under immunomodulatory or antimicrobial therapy may also have normal concentrations, even if an infection is present. On the other hand, the elevation of these serum markers cannot confirm an infection and, therefore, cannot be recommended as stand-alone test for diagnosing PJI. The high false-positive rate can be explained by the fact that they are systemic parameters influenced by other inflammatory conditions, such as autoimmune disorders (i.e. rheumatoid arthritis, psoriasis, or systemic lupus erythematosus), active cancer, or other infectious foci at another site (i.e. pneumonia or endocarditis). Due to these limitations, we propose that the role of serum inflammatory markers is to supplement other diagnostic tests, rather than to be used in isolation. More invasive investigations are needed to accurately diagnose PJI. We recommend obtaining synovial fluid analysis in all cases with suspected PJI, even if CRP, IL-6, and fibrinogen are normal.

The majority of included studies ( n= 65/89, 73 %) were of low or very low quality. Only 24 studies (27 %) were of moderate quality and none were of high quality. Significant limitations of this meta-analysis include the different infection definitions utilized, the heterogeneity of inclusion and exclusion criteria, the wide variability in applied cutoffs, incorporation bias (for CRP, ESR, and D-dimer), high between-study heterogeneity across nearly all performance measures of all markers, and limited information on test reproducibility. Almost all studies failed to differentiate between the performance of serum inflammatory parameters in acute (early postoperative/early acute and acute haematogenous/late acute) vs. chronic infections and were limited to hip and knee PJI. Therefore, based on these data, it is unclear whether our recommendations can be generalized to periprosthetic shoulder infections (or other less common types of arthroplasty), in which the epidemiology and causative microorganisms differ. Lastly, some studies included not only patients with osteoarthritis but also those with rheumatoid arthritis and/or immunosuppression. However, due to the limited number of such cases, stratified analysis was not feasible.

## 5 Conclusion

Although serum CRP, fibrinogen, and interleukin-6 demonstrated the highest diagnostic performance among the analysed markers in our meta-analysis, their accuracy remains insufficient to recommend their use as stand-alone tests for diagnosing PJI. These markers may be useful as part of a broader diagnostic workup but should be interpreted in conjunction with other clinical and laboratory findings.

In light of these findings, future PJI definitions may consider placing less emphasis on serum biomarkers as primary diagnostic criteria, favouring more accurate modalities such as synovial fluid analysis. Nevertheless, serum biomarkers might retain value as adjunctive screening tools, particularly in early diagnostic stages. Importantly, these results support pursuing synovial fluid analysis regardless of normal serum marker levels.

## Supplement

10.5194/jbji-10-363-2025-supplement The supplement related to this article is available online at https://doi.org/10.5194/jbji-10-363-2025-supplement.

## Data Availability

All data generated or analysed in this position paper are included in the published article or in the Supplement.

## Team list

Marjan Wouthuyzen-Bakker (Department of Medical Microbiology and Infection Prevention, University Medical Center Groningen, University of Groningen, Groningen, the Netherlands), Thomas Bauer (Department of Pathology, the Cleveland Clinic Foundation, Cleveland, OH, USA), Elie Berbari (Division of Public Health, Infectious Diseases and Occupational Medicine, Department of Medicine, Mayo Clinic College of Medicine and Science, Mayo Clinic, Rochester, MN, USA), Martin Clauss (Department of Orthopaedics and Trauma Surgery, University Hospital Basel, Basel, Switzerland), Nicolas Cortes-Penfield (Division of Infectious Diseases, University of Nebraska Medical Center, Omaha, NE, USA), Matthew Dietz (Department of Orthopaedics, School of Medicine, West Virginia University, Morgantown, WV, USA), Jaime Esteban (Department of Medical Microbiology, IIS-Fundacion Jimenez Diaz, Madrid, Spain), Tristan Ferry (Infectious Diseases Department, Croix-Rousse Hospital, Hospices Civils de Lyon, Lyon, France), Thorsten Gehrke (Department of Orthopaedic Surgery, ENDO-Klinik Hamburg, Specialist Clinic for Bone and Joint Surgery, Hamburg, Germany), Andor Glaudemans (Medical Imaging Center, Department of Nuclear Medicine & Molecular Imaging, University Medical Center Groningen, University of Groningen, Groningen, the Netherlands), Benjamin Langworth (Division of Biostatistics and Health Data Science, School of Public Health, University of Minnesota, Rochester, MN, USA), Martin McNally (the Bone Infection Unit, Nuffield Orthopaedic Centre, Oxford University Hospitals, Oxford, UK), Andy Miller (Department of Infectious Disease, Hospital for Special Surgery, New York, NY, USA), Sandra Nelson (Division of Infectious Diseases, Massachusetts General Hospital, Harvard Medical School, Boston, MA, USA), Javad Parvizi (Department of Orthopaedic Surgery, International Joint Center, Acibadem University Hospital, Istanbul, Türkiye), Robin Patel (Division of Infectious Diseases, Department of Internal Medicine, Mayo Clinic, Rochester, MN, USA), Holger Rohde (Institute of Medical Microbiology, Virology and Hygiene, University Medical Center Hamburg-Eppendorf, Hamburg, Germany), Thorsten Seyler (Department of Orthopaedics, Duke University Hospital, Durham, NC, USA), Irene Sigmund (Department of Orthopaedics and Trauma Surgery, Medical University of Vienna, Vienna, Austria), Alex Soriano (Department of Infectious Diseases, University of Barcelona, Institut d'Investigacions Biomèdiques August Pi i Sunyer, Hospital Clinic of Barcelona, CIBERINF Ciber in Infectious Diseases, Barcelona, Spain), and Ricardo Sousa (Department of Orthopedics, Centro Hospitalar Universitário de Santo António, Porto, Portugal) on behalf of the “Serum Marker Workgroup” for the Unified PJI Definition Taskforce.
