# Supplementary material for: Serum inflammatory markers for the screening and diagnosis of periprosthetic joint infection: a systematic review and meta-analysis
Source: J Bone Jt Infect. 2025 Oct 10;10(5):363–76. doi: 10.5194/jbji-10-363-2025 (PMC12604141; doi:10.5194/jbji-10-363-2025)
Supplement: The supplement related to this article is available online at https://doi.org/10.5194/jbji-10-363-2025-supplement. [file jbji-10-363-2025-supplement.pdf]

Supplement of J. Bone Joint Infect., 10, 363–376, 2025
https://doi.org/10.5194/jbji-10-363-2025-supplement
© Author(s) 2025. CC BY 4.0 License.

Supplement of

Serum inflammatory markers for the screening and diagnosis of periprosthetic joint infection: a systematic review and meta-analysis

Irene K. Sigmund et al.

Correspondence to: Irene K. Sigmund (irene.sigmund@meduniwien.ac.at)

The copyright of individual parts of the Supplement might differ from the article licence.

Search Queries for PubMed (MEDLINE)	Results
("Arthroplasty"[Mesh] OR "Joint Prosthesis"[Mesh] OR “Periprosthetic joint infection*”[tiab] OR “Prosthetic joint infection*”[tiab] OR “PJI”[tiab] OR “Arthroplast*”[tiab] OR “Total joint*”[tiab] OR “Joint prosthe*”[tiab] OR “DAIR”[tiab] OR “debridement*”[tiab]) AND (“fibrinogen” OR “neutrophil ratio” OR “lymphocyte ratio” OR “monocyte ratio” OR “globulin ratio” OR “platelet volume” OR "sedimentation rate" OR "Blood Sedimentation"[Mesh] OR ESR OR "c-reactive protein" OR "c reactive protein" OR CRP OR "C-Reactive Protein"[Mesh] OR d-dimer OR IL-6 OR "interleukin 6" OR PCT OR procalcitonin OR "Procalcitonin"[MESH]) AND ("Sensitivity and Specificity"[Mesh] OR “sensitiv*”[tiab] OR “specificit*”[tiab] OR “accura*”[tiab] OR “positive predictive value*”[tiab] OR “negative predictive value*”[tiab] OR “PPV” [tiab] OR “NPV” [tiab])	802
**Search queries for EMBASE**	
("Arthroplasty"[Mesh] OR "Joint Prosthesis"[Mesh] OR “Periprosthetic joint infection*”[tiab] OR “Prosthetic joint infection*”[tiab] OR “PJI”[tiab] OR “Arthroplast*”[tiab] OR “Total joint*”[tiab] OR “Joint prosthe*”[tiab] OR “DAIR”[tiab] OR “debridement*”[tiab]) AND (“fibrinogen” OR “neutrophil ratio” OR “lymphocyte ratio” OR “monocyte ratio” OR “globulin ratio” OR “platelet volume” OR "sedimentation rate" OR "Blood Sedimentation"[Mesh] OR ESR OR "c-reactive protein" OR "c reactive protein" OR CRP OR "C-Reactive Protein"[Mesh] OR d-dimer OR IL-6 OR "interleukin 6" OR PCT OR procalcitonin OR "Procalcitonin"[MESH]) AND ("Sensitivity and Specificity"[Mesh] OR “sensitiv*”[tiab] OR “specificit*”[tiab] OR “accura*”[tiab] OR “positive predictive value*”[tiab] OR “negative predictive value*”[tiab] OR “PPV” [tiab] OR “NPV” [tiab])	1222

Table S1. Search queries for PubMed (MEDLINE) and EMBASE.

**Study (Year)**	**PJI reference standard**	**N° Patients**	**N° PJIs**	**N° AF**	**N° knees**	**N° hips**	**N° shoulders**	**Cutoff (mg/L)**	**Sensitivity (95%CI)**	**Specificity (95%CI)**	**PPV (95%CI)**	**NPV (95%CI)**
Abou El-Khier 2013	Clinical criteria / Other	40	11	29	14	26	0	18	100 (na)	86.2 (na)	68.8 (na)	100 (na)
Ackmann 2020	ICM 2018	119	52	67	71	48	0	12	88 (na)	87 (na)	84 (na)	91 (na)
Ahmadi 2018	Clinical outcome / Other	537	169	368	0	0	537	10	21.1 (na)	79.4 (na)	37.5 (na)	63.1 (na)
Alijanipour 2013	MSIS 2013	1949	260	1689	755	1194	0	10 (hips)	88.1 (na)	77.4 (na)	18.6 (na)	99.1 (na)
Alijanipour 2013	MSIS 2013	1949	260	1689	755	1194	0	10 (knees)	96.6 (na)	70.2 (na)	43 (na)	98.9 (na)
Austin 2008	Clinical outcome / Other	296	116	180	296	0	0	10	94 (na)	74 (na)	70 (na)	95 (na)
Bare 2006	Clinical outcome / Other	295	79	216	295	0	0	10	60 (na)	63 (na)	45 (na)	76 (na)
Berger 2017	MSIS 2013	121	34	87	85	36	0	10	70.6 (52.2 -84.9)	81.6 (71.9-89.1)	60 (43.3-75.1)	87.6 (na)
Bernard 2004	Clinical outcome / Other	230	209	21	42	167	0	10	97 (na)	81 (na)	98 (na)	84 (na)
Bin 2020	MSIS 2013	90	53	37	33	57	0	4.93	94.34 (na)	72.97 (na)	83.3 (na)	90 (na)
Bottner 2007	Clinical criteria / Other	78	21	57	28	50	0	15	95 (na)	91 (na)	80 (na)	98 (na)
Buttaro 2010	Clinical criteria / Other	69	11	58	0	69	0	10	72 (41 - 100)	91 (83-9)	61 (31-91)	94 (87-100)
Cao 2020	MSIS 2013	214	77	137	na	na	na	10.9	79.92 (na)	75.91 (na)	64.21 (na)	86.55 (na)
Chen Xi 2021	Clinical outcome / Other	75	25	50	40	35	0	12.6	92 (na)	94 (na)	88.5 (na)	95.9 (na)
Chisari 2021	ICM 2018	259	78	181	226	33	0	10	83.3 (na)	91.2 (na)	83.3 (na)	91.2 (na)
Cipriano 2012	Clinical outcome / Other	61	19	42	21	40	0	17 (with inflammatory arthritis)	93.8 (87-100)	70.3 (58-83)	58 (44-71)	96 (91-100)
Cipriano 2012	Clinical outcome / Other	839	146	693	362	477	0	15 (without)	85.8 (83-88)	83.4 (81-86)	56 (52-60)	96 (95-97)
Claasen 2016	Clinical outcome / Other	34	8	26	34	0	0	3	38.0 (9-76)	73 (52-88)	30 (7-65)	79 (58-93)
Deirmengian 2010	Clinical outcome / other	51	14	37	22	29	0	10	71 (na)	86 (na)	67 (na)	89 (na)
Deirmengian 2021	MSIS 2013	305	57	248	203	102	0	10	92.2 (81.1-97.8)	83.1 (76.7-88.3)	61 (52.8-68.7)	97.4 (93.5-99)
Della Valle 2007	Clinical outcome / other	94	41	53	94	0	0	10	95.1 (na)	75 (na)	75 (na)	95.2 (na)
Denyer 2023	IDSA 2013	283	124	159	283	0	0	10	58.62 (na)	98.3 (na)	90.67 (na)	67.12 (na)
Di Cesare 2005	Clinical criteria / Other	58	17	41	na	na	na	10	94 (na)	78 (na)	64 (na)	97 (na)
Ding 2019	MSIS 2013	70	19	51	na	na	0	10	94.7 (74.0-99.9)	77.6 (63.4-88.2)	62.1 (49-73.6)	97.4 (84.9-99.6)
Dong 2024	MSIS 2013	182	64	118	61	121	0	5.8	81.6 (na)	78.3 (na)	86.1 (na)	72 (na)
Elgeidi 2014	Clinical criteria / Other	40	11	29	14	26	0	18	100 (na)	86.2 (na)	68.8 (na)	100 (na)
Erdemli 2018	MSIS 2013	88	36	52	59	29	0	4.1	72.22 (54.8-85.8)	65.38 (50.9-78)	59.1 (na)	77.3 (na)
Ettinger 2015	MSIS 2011	98	41	57	39	57	2	3.0	80 (na)	64 (na)	37.2 (na)	92.3 (na)
Fernandez-Sampedro 2017	Clinical outcome / Other	498	130	368	46	84	0	10	70.3 (61.6 - 78.1)	84.5 (80.4-88)	61.2 (52.8-69.1)	89.1 (85.3-92.2)
Fernandez-Sampedro 2022	IDSA 2013	180	39	141	62	118	0	10	84.6 (73-96)	82.1 (76-88)	56.8 (44-69)	95 (87-100)
Fink 2008	Clinical / other	145	40	105	145	0	0	14	72.5 (58.7-86.3)	80.9 (73.4-88.4)	59.2 (45.4-73)	88.5 (81-96)
Fink 2013	Clinical / other	100	45	55	0	100	0	10	64 (50-78)	75 (63-86)	67 (53-81)	71 (60-84)
Fink 2018	Clinical / other	116	27	89	116	0	0	10	59.3 (na)	82 (na)	50 (na)	86.9 (na)
Fink 2020	Clinical / other	390	180	210	na	na	0	10	57.2 (na)	79 (na)	70.1 (na)	68.3 (na)
Fu 2019	MSIS 2011	30	15	15	27	3	0	10	80 (51.9-95.7)	80 (51.9-95.7)	80 (51.9-95.7)	80 (51.9-95.7)
Ghanem 2009	Clinical / other	479	127	352	0	479	0	10	91.1 (85-95)	76.6 (72-81)	74.5 (66-81)	94.8 (92-97)
Greidanus 2007	Clinical / other	151	45	106	151	0	0	14	91 (82-99)	86 (80-93)	74 (63-86)	95 (91-99)
Grzelecki 2021	ICM 2018	133	68	65	72	61	0	10	85.3 (na)	90.8 (na)	90.6 (na)	85.5 (na)
Hu 2020	MSIS 2013	77	40	37	14	61	0	10	80 (na)	78.38 (na)	80 (na)	78.38 (na)
Huang 2022	MSIS 2013	99	43	56	51	48	0	18	62.79 (46.7-77.0)	87.5 (75.9-94.8)	79.4 (65-88.9)	75.4 (67-82.1)
Itasaka 2001	Clinical / other	48	6	42	0	46	0	3	83 (na)	76 (na)	33 (na)	97 (na)
Klemt 2023	EBJIS 2021	464	191	273	0	464	0	10	81.2 (na)	75.8 (na)	70.1 (na)	85.2 (na)
Klim 2024	EBJIS 2021	67	43	24	55	11	1	36	76 (na)	71 (na)	86 (na)	55 (na)
Kuo 2018	ICM 2013	214	25	189	131	83	0	11.5	84.6 (65.1-95.6)	78.3 (71.44-84.15)	36.7 (29.5-44.5)	97.2 (93.3-98.8)
Kuo 2022	ICM 2018	76	42	34	41	35	0	10	93 (na)	47 (na)	68 (na)	84 (na)

Levent 2021	ICM 2018	259	108	151	143	116	0	10	79,8 (na)	76.8 (na)	71.3 (na)	84.1 (na)
Li 2019	ICM 2014	565	95	470	153	412	0	9.0	72.4 (na)	84.3 (na)	49.1 (na)	93.6 (na)
Liu 2014	MSIS 2011	102	73	29	102	0	0	10 (non-obese)	95.8 (na)	64.2 (na)	82.1 (na)	90 (na)
Liu 2014	MSIS 2011	102	73	29	102	0	0	10 (obese)	95.2 (na)	46.1 (na)	74.1 (na)	85.7 (na)
Liu 2022	MSIS 2011	58	38	20	51	7	0	19.2	57.9 (43.5-67.1)	80 (71.1-89.7)	84.6 (76.1-92.3)	50 (41.7-60.2)
Maimaiti 2021	MSIS 2013	246	125	121	99	147	0	7.3	75.2 (na)	84.3 (na)	83.2 (na)	76.7 (na)
Majors 2019	ICM 2018	59	32	27	59	0	0	17	67 (41-86)	67 (43-85)	63 (39-83)	70 (46-87)
Muñoz-Mahamud 2022	ICM 2018	93	24	69	66	27	0	19.5	61 (na)	90 (na)	68 (na)	87 (na)
Nilsdotter-Augustinsson 2007	Clinical	71	25	46	na	na	na	10	82 (na)	71 (na)	53 (na)	91 (na)
Parvizi 2012	Clinical	55	25	30	55	0	0	16.5	76 (na)	93.3 (na)	90.5 (na)	82 (na)
Paziuk 2020	ICM 2018	4938	949	3989	na	na	0	15	87.5 (85.2-89.6)	74.3 (72.4-76.1)	59 (na)	93 (na)
Piper 2010	Clinical	582	135	447	297	221	64	10 (hip)	74 (na)	78 (na)	38 (na)	94 (na)
Piper 2010	Clinical	582	135	447	297	221	64	10 (knee)	83 (na)	79 (na)	60 (na)	92 (na)
Piper 2010	Clinical	582	135	447	297	221	64	10 (shoulder)	42 (na)	84 (na)	53 (na)	78 (na)
Qin 2020 BJR	MSIS 2013	93	37	56	44	48	0	10	94.6 (81.8-99.3)	35.7 (49.6-81.8)	49.3 (na)	90.9 (na)
Qin 2020 JoA	MSIS 2013	122	55	67	44	78	0	7.5	81.1 (68-90.6)	65.7 (53.1-76.8)	65.2 (na)	81.5 (na)
Schinsky 2008	Clinical outcome	235	55	180	0	235	0	10	94 (87-100)	71 (64-79)	96 (49-69)	96 (92-100)
Shah 2016	MSIS 2013	121	14	107	24	97	0	10	71 (na)	44 (na)	19 (na)	89 (na)
Shahi 2017	ICM 2014	143	57	86	75	68	0	10	79 (66-88)	80 (72-86)	61 (na)	90 (na)
Shang 2022	MSIS 2013	206	79	127	117	110	0	8.2	83.5 (na)	85 (na)	77.6 (na)	89.3 (na)
Shi 2023	ICM 2018	244	87	157	78	166	0	8.4	86 (na)	91.4 (na)	83.33 (na)	92 (na)
Sigmund 2021	EBJIS 2021	177	75	102	86	91	0	10	68 (56.7-77.5)	87.1 (79-92.4)	79.7 (69.8-89.5)	78.6 (71-86.2)
Tetreault 2014	modified MSIS 2011	119	32	87	60	59	0	11.2	97 (94-100)	76 (68-84)	60 (51-68)	99 (96-100)
Tirumala 2021	ICM 2018	538	206	332	538	0	0	10	76.5 (na)	77.7 (na)	65.3 (na)	79.4 (na)
Tohtz 2010	Clinical criteria / Other	64	19	45	0	64	0	10	57.9 (na)	80 (na)	55 (na)	81.8 (na)
Villacis 2014	Clinical criteria / Other	34	14	20	0	0	34	10	0 (na)	95 (na)	50 (na)	59 (na)
Wang 2020	MSIS 2013	157	51	106	34	123	0	5	92.2 (na)	81.1 (na)	70.2 (na)	95.6 (na)
Wang 2021	MSIS 2013	93	37	56	45	48	0	13	89.2 (75-97)	50 (36-64)	54.1 (na)	87.5 (na)
Wang R 2023	MSIS 2013	162	56	106	54	108	0	8.3	78.6 (66-88)	88.7 (81-94)	78.6 (68-87)	88.7 (na)
Wang X 2023	ICM 2018	253	116	137	97	156	0	11	82.8 (na)	86.9 (na)	94.5 (na)	73.9 (na)
Worthington 2010	Clinical criteria / Other	46	16	30	0	46	0	10	94 (na)	97 (na)	94 (na)	50 (na)
Wouthuyzen-Bakker 2018	MSIS 2011	52	15	37	17	32	3	10	66.7 (39-88)	70.4 (50-86)	55.6 (39-71)	79.2 (64-89)
Wu 2014	Clinical criteria / Other	156	37	119	16	140	0	10	92 (na)	69 (na)	39 (na)	97 (na)
Wu 2020	MSIS 2013	116	81	35	48	71	0	10.8	72.7 (na)	95.1 (na)	80 (na)	91.5 (na)
Wu 2023	MSIS 2013	455	187	268	111	344	0	7.2	80.2 (na)	84.7 (na)	78.5 (na)	85.9 (na)
Xu 2019	ICM 2014	318	129	189	65	253	0	7.4	79.1 (na)	84.7 (na)	77.9 (na)	85.6 (na)
Xu 2020	MSIS 2013	360	153	207	55	305	0	8.5	78.4 (na)	89.9 (na)	85.1 (na)	84.9 (na)
Xu 2021	MSIS 2013	65	34	31	na	na	0	10	82.4 (na)	77.4 (na)	80 (na)	80 (na)
Xu 2022	MSIS 2013	543	245	298	na	na	0	7.4	79.1 (na)	86 (na)	82.3 (na)	83.4 (na)
Yang 2021	ICM 2018	156	57	99	50	106	0	12.5	91.2 (na)	82.7 (na)	87 (na)	90.2 (na)
Ye 2021	ICM 2018	158	54	104	46	112	0	7	72.2 (na)	82.7 (na)	68.4 (na)	85.1 (na)
Yin 2021	MSIS 2011	35	15	20	16	19	0	11.2	67(na)	85 (na)	77 (na)	77 (na)
Yu 2020	MSIS 2013	121	20	101	93	28	0	9.3	70 (na)	79.2 (na)	40 (na)	93 (na)
Yu 2021	modified ICM 2014	139	62	77	95	44	0	9.8	72.6 (na)	80.5 (na)	75 (na)	78.5 (na)

Table S2. Characteristics of all included studies evaluating serum C-reactive protein (CRP).

PJI: periprosthetic joint infection, AF: aseptic failure, CI: confidence interval.

Study (Year)	PJI reference standard	N° Patients	N° PJIs	N° AF	N° knees	N° hips	N° shoulders	Cutoff (mm/h)	Sensitivity (95%CI)	Specificity (95%CI)	PPV (95%CI)	NPV (95%CI)
Abou El-Khier 2013	Clinical criteria / Other	40	11	29	14	26	0	45	81.8 (na)	82.8 (na)	64.3 (na)	92.3 (na)
Ahmadi 2018	Clinical outcome / Other	537	169	368	0	0	537	22	21.5 (na)	75.9 (na)	33.3 (na)	63.3 (na)
Alijanipour 2013	MSIS 2013	1949	260	1689	755	1194	0	30 (hips)	94.7 (na)	71.2 (na)	14.7 (na)	99.6 (na)
Alijanipour 2013	MSIS 2013	1949	260	1689	755	1194	0	30 (knees)	84.1 (na)	68.3 (na)	37.3 (na)	98.3 (na)
Austin 2008	Clinical outcome / Other	296	116	180	296	0	0	30	91 (na)	72 (na)	68 (na)	93 (na)
Bare 2006	Clinical outcome / Other	295	79	216	295	0	0	30	63 (na)	55 (na)	39 (na)	77 (na)
Berger 2017	MSIS 2013	121	34	87	85	36	0	30	47.6 (25.7-70.2)	82.8 (70.6-91.4)	50 27.2-72.8)	81.4 (69.1-90.3)
Bernard 2004	Clinical outcome / Other	230	209	21	42	167	0	30	87 (na)	47 (na)	94 (na)	62 (na)
Bottner 2007	Clinical criteria / Other	78	21	57	28	50	0	32	81 (na)	89 (na)	74 (na)	93 (na)
Buttaro 2010	Clinical criteria / Other	69	11	58	0	69	0	na	72 (41-100)	86 (76-95)	50 (22-77)	94 (84-100)
Chen Xi 2021	Clinical outcome / Other	75	25	50	40	35	0	27	88 (na)	80 (na)	68.7 (na)	93 (na)
Chen Y 2022	MSIS 2013	52	26	26	25	27	0	32	73.08 (na)	76.92 (na)	76 (na)	74.07 (na)
Chisari 2021	ICM 2018	259	78	181	226	33	0	36	72.6 (na)	87.8 (na)	75.7 (na)	85.9 (na)
Cipriano 2012	Clinical outcome / Other	61	19	42	21	40	0	30 (with inflammatory arthritis)	94.4 (88-100)	59.4 (46-72)	53 (40-66)	96 (90-100)
Cipriano 2012	Clinical outcome / Other	839	146	693	362	477	0	32 (without)	87.2 (85-90)	67.1 (64-71)	40 (36-43)	96 (94-97)
Deirmengian 2010	Clinical outcome / other	51	14	37	22	29	0	30	86 (na)	73 (na)	55 (na)	93 (na)
Deirmengian 2021	MSIS 2013	305	57	248	203	102	0	30	88.2 (76.1-95.6)	84.8 (78.6-89.7)	62.5 (53.7-70.5)	96.2 (92.2-98.2)
Denyer 2023	IDSA 2013	283	124	159	283	0	0	30	87.3 (na)	78.1 (na)	81.6 (na)	85.42 (na)
Della Valle 2007	Clinical outcome / other	94	41	53	94	0	0	30	90.2 (na)	66 (na)	67.3 (na)	89.7 (na)
Di Cesare 2005	Clinical criteria / Other	58	17	41	na	na	na	30	100 (na)	56 (na)	49 (na)	100 (na)
Ding 2019	MSIS 2013	70	19	51	na	na	0	30	100 (66.4-100)	54.6 (36.4-71.9)	37.5 (29.2-46.6)	100 (na)
Dong 2024	MSIS 2013	182	64	118	61	121	0	20.5	92.1 (na)	69.6 (na)	83.3 (na)	84.2 (na)
Elgeidi 2014	Clinical criteria / Other	40	11	29	14	26	0	45	81.8 (na)	82.8 (na)	64.3 (na)	92.3 (na)
Fernandez-Sampedro 2022	IDSA 2013	180	39	141	62	118	0	15	82 (70-94)	72.8 (65-80)	45.7 (34-57.4)	93.5 (82.8-100)
Fu 2019	MSIS 2011	30	15	15	27	3	0	30	33.3 (11.8-61.6)	100 (78.2-100)	100 (47.8-100)	60 (38.7-78.9)
Ghanem 2009	Clinical / other	479	127	352	0	479	0	30	94.3 (89-98)	70.2 (65-75)	55.5 (48-62)	96.9 (94-99)
Greidanus 2007	Clinical / other	151	45	106	151	0	0	22.5	93 (86-100)	83 (76-90)	71 (59-82)	96 (93-100)
Grzelecki 2021	ICM 2018	133	68	65	72	61	0	30	73.5 (na)	86.2 (na)	84.7 (na)	75.7 (na)
Hu 2020	MSIS 2013	77	40	37	14	61	0	30	82.5 (na)	64.9 (na)	71.74 (na)	77.42 (na)
Huang 2022	MSIS 2013	99	43	56	51	48	0	34	58.14 (42.1-73)	83.93 (71.7-92.4)	73.5 (59.2-84.2)	72.3 (64.3-79.1)
Itasaka 2001	Clinical / other	48	6	42	0	46	0	30	67 (na)	74 (na)	27 (na)	94 (na)
Klemt 2023	EBJIS 2021	464	191	273	0	464	0	30	67 (na)	78.8 (na)	68.8 (na)	77.3 (na)
Kuo 2018	MSIS	214	25	189	131	83	0	28.5	88 (68.8-97.4)	65.1 (57.5-72.2)	26.8 (22.2-32)	97.4 (92.8-99.1)
Kuo 2022	ICM 2018	76	42	34	41	35	0	30	74 (na)	82 (na)	82 (na)	73 (na)
Li 2019	ICM 2014	565	95	470	153	412	0	26.5	63.2 (na)	85.7 (na)	48 (na)	91.7 (na)
Liu 2014	MSIS	102	73	29	102	0	0	30 (non-obese)	91.3 (na)	80 (na)	87.5 (na)	85.7 (na)
Liu 2014	MSIS	102	73	29	102	0	0	30 (obese)	94.8 (na)	61.5 (na)	78.7 (na)	88.9 (na)
Liu 2022	MSIS 2011	58	38	20	51	7	0	24.5	76.3 (62.3-82.2)	93.5 (88.5-100)	93.5 (84.3-98.9)	66.7 (54.3-77.7)
Maimaiti 2021	MSIS 2013	246	125	121	99	147	0	26	69.6 (na)	88.4 (na)	86.1 (na)	73.8 (na)
Majors 2019	ICM 2018	59	32	27	59	0	0	27	70 (46-87)	64 (41-82)	64 (41-82)	70 (46-87)
Muñoz-Mahamud 2022	ICM 2018	93	24	69	66	27	0	20	74 (na)	82 (na)	50 (na)	89 (na)

Nilsdotter-Augustinsson 2007	Clinical	71	25	46	na	na	na	30	64 (na)	87 (na)	67 (na)	86 (na)
Paziuk 2020	ICM 2018	4938	949	3989	na	na	0	46	78.6 (75.8-81.2)	77.7 (76.2-79.2)	52 (na)	92 (na)
Piper 2010	Clinical	582	135	447	297	221	64	30 (hip)	47 (na)	84 (na)	36 (na)	90 (na)
Piper 2010	Clinical	582	135	447	297	221	64	30 (knee)	71 (na)	89 (na)	71 (na)	89 (na)
Qin 2020 BJR	MSIS 2013	93	37	56	44	48	0	30	54.1 (36.9-70.5)	58.9 (45-71.9)	46.5 (na)	67.4 (na)
Qin 2020 JoA	MSIS 2013	122	55	67	44	78	0	41	63.6 (49.6-76.2)	70.2 (57.7-80.7)	63.6 (na)	70.1 (na)
Schinsky 2008	Clinical outcome	235	55	180	0	235	0	30	97 (93-100)	39 (31-47)	42 (34-50)	96 (92-100)
Shah 2016	MSIS 2013	121	14	107	24	97	0	30	86 (na)	40 (na)	20 (na)	94 (na)
Shahi 2017	ICM 2014	143	57	86	75	68	0	30	73 (60-84)	78 (70-85)	58 (na)	88 (na)
Shang 2022	MSIS 2013	206	79	127	117	110	0	15.7	88.6 (na)	75.4 (na)	70 (na)	91.5 (na)
Shi 2023	ICM 2018	244	87	157	78	166	0	26.5	80.2 (na)	90.1 (na)	82.14 (na)	88.16 (na)
Tirumala 2021	ICM 2018	538	206	332	538	0	0	30	81.6 (na)	77.1 (na)	68.9 (na)	87.1 (na)
Tohtz 2010	Clinical criteria / Other	64	19	45	0	64	0	30	73.7 (na)	88.9 (na)	73.7 (na)	88.9 (na)
Villacis 2014	Clinical criteria / Other	34	14	20	0	0	34	30	21 (na)	65 (na)	30 (na)	54 (na)
Wang 2020	MSIS 2013	157	51	106	34	123	0	24.5	82.4 (na)	77.4 (na)	63.6 (na)	90.1 (na)
Wang 2021	MSIS 2013	93	37	56	45	48	0	34	54.1 (37-71)	78.6 (66-88)	62.5 (na)	72.1 (na)
Wang R 2023	MSIS 2013	162	56	106	54	108	0	32	75 (62-86)	85.9 (78-92)	73.7 (63-82)	86.7 (82-91)
Wang X 2023	ICM 2018	253	116	137	97	156	0	36.5	69 (na)	84.7 (na)	82.4 (na)	74.7 (na)
Worthington 2010	Clinical criteria / Other	46	16	30	0	46	0	30	81 (na)	100 (na)	100 (na)	39 (na)
Wouthuyzen-Bakker 2018	MSIS 2011	52	15	37	17	32	3	30	72.7 (39-94)	69.6 (47-87)	53.3 (36-70)	84.2 (66-94)
Wu 2014	Clinical criteria / Other	156	37	119	16	140	0	30	75 (na)	69 (na)	35 (na)	93 (na)
Wu 2020	MSIS 2013	116	81	35	48	71	0	29	69.7 (na)	92.2 (na)	74.1 (na)	90.4 (na)
Wu 2023	MSIS 2013	455	187	268	111	344	0	33.5	67.4 (na)	75.7 (na)	65.9 (na)	76.8 (na)
Xu 2019	ICM 2014	318	129	189	65	253	0	42.5	69 (na)	81.5 (na)	72.4 (na)	79.4 (na)
Xu 2020	MSIS 2013	360	153	207	55	305	0	40.5	66.7 (na)	86.5 (na)	78.5 (na)	77.8 (na)
Xu 2021	MSIS 2013	65	34	31	na	na	0	30	82.4 (na)	67.7 (na)	73.7 (na)	77.8 (na)
Xu 2022	MSIS 2013	543	245	298	na	na	0	42.5	65.5 (na)	84.2 (na)	77.3 (na)	74.8 (na)
Yang 2021	ICM 2018	156	57	99	50	106	0	36.5	70.2 (na)	85.9 (na)	74.1 (na)	83.3 (na)
Ye 2021	ICM 2018	158	54	104	46	112	0	43	68.5 (na)	81.7 (na)	66.1 (na)	83.3 (na)
Yu 2020	MSIS 2013	121	20	101	93	28	0	22	63.2 (na)	74 (na)	31.6 (na)	91.4 (na)

Table S3. Characteristics of all included studies evaluating serum erythrocyte sedimentation rate (ESR).

PJI: periprosthetic joint infection, AF: aseptic failure, CI: confidence interval.

Study (Year)	PJI reference standard	N° Patients	N° PJIs	N° AF	N° knees	N° hips	N° shoulders	Cutoff (G/L)	Sensitivity (95%CI)	Specificity (95%CI)	PPV (95%CI)	NPV (95%CI)
Abou El-Khier 2013	Clinical criteria / Other	40	11	29	14	26	0	9.2	90.9 (na)	75.9 (na)	58.8 (na)	95.6 (na)
Bottner 2007	Clinical criteria / Other	78	21	57	28	50	0	11	70 (na)	60 (na)	40 (na)	86 (na)
Di Cesare 2005	Clinical criteria / Other	58	17	41	na	na	na	11	47 (na)	100 (na)	100 (na)	82 (na)
Elgeidi 2014	Clinical criteria / Other	40	11	29	14	26	0	9.2	90 (na)	75.9 (na)	58.8 (na)	95.6 (na)
Itasaka 2001	Clinical / other	48	6	42	0	46	0	9	33 (na)	91 (na)	33 (na)	91 (na)
Klim 2024	EBJIS 2021	67	43	24	55	11	1	9.6	51 (na)	76 (na)	84 (na)	39 (na)
Li 2019	ICM 2014	565	95	470	153	412	0	6.2	59.2 (na)	59.8 (na)	23.6 (na)	87.5 (na)
Maimaiti 2021	MSIS 2013	246	125	121	99	147	0	7.55	32.8 (na)	87.6 (na)	73.2 (na)	55.8 (na)
Sigmund 2021	EBJIS 2021	177	75	102	86	91	0	10	36 (26.1-47.3)	89.1 (81.3-93.9)	71.1 (56.6-85.5)	65.2 (57.3-73.2)
Tohtz 2010	Clinical criteria / Other	64	19	45	0	64	0	10	21.1 (na)	91.1 (na)	50 (na)	73.2 (na)
Toossi 2012	Clinical criteria / Other	1856	751	1105	881	975	0	7.8	55.4 (52-59)	66.2 (63-69)	52.6 (49-56)	68.5 (66-71)
Villacis 2014	Clinical criteria / Other	34	14	20	0	0	34	11	7 (na)	95 (na)	50 (na)	59 (na)
Yang 2021	ICM 2018	156	57	99	50	106	0	7.4	78.8 (na)	49.2 (na)	57.1 (na)	72.9 (na)
Yu 2020	MSIS 2013	121	20	101	93	28	0	8.91	35 (na)	94.1 (na)	53.8 (na)	88 (na)

Table S4. Characteristics of all included studies evaluating serum white blood cell count (WBC).

PJI: periprosthetic joint infection, AF: aseptic failure, CI: confidence interval.

Study (Year)	PJI reference standard	N° Patients	N° PJIs	N° AF	N° knees	N° hips	N° shoulders	Cutoff (g/L)	Sensitivity (95%CI)	Specificity (95%CI)	PPV (95%CI)	NPV (95%CI)
Bin 2020	MSIS 2013	90	53	37	33	57	0	3.6	79.25 (na)	94.59 (na)	95.5 (na)	76.1 (na)
Chen Xi 2021	Clinical outcome / Other	75	25	50	40	35	0	3.7	92 (na)	84 (na)	75.8 (na)	91.2 (na)
Chen Y 2022	MSIS 2013	52	26	26	25	27	0	3.3	80.77 (na)	73.08 (na)	75 (na)	79.17 (na)
Chisari 2021	ICM 2018	259	78	181	226	33	0	3.9	66.7 (na)	91.7 (na)	80 (na)	84.6 (na)
Dong 2024	MSIS 2013	182	64	118	61	121	0	3.6	71.1 (na)	73.9 (na)	81.8 (na)	60.7 (na)
Li 2019	ICM 2014	565	95	470	153	412	0	4.0	76.3 (na)	86.2 (na)	53.7 (na)	94.6 (na)
Maimaiti 2021	MSIS 2013	246	125	121	99	147	0	4.1	75.2 (na)	86.8 (na)	85.5 (na)	77.2 (na)
Sigmund 2021	EBJIS 2021	177	75	102	86	91	0	4.6	68.5 (57.1-78)	88.5 (80.4-93.6)	82 (72.3-91.6)	78.7 (na)
Wang 2020	MSIS 2013	157	51	106	34	123	0	3.6	86.3 (na)	84 (na)	72.1 (na)	92.7 (na)
Wu 2020	MSIS 2013	116	81	35	48	71	0	3.6	75.8 (na)	86.4 (na)	64.1 (na)	91.8 (na)
Wu 2023	MSIS 2013	455	187	268	111	344	0	3.7	54 (na)	84.3 (na)	71.1 (na)	72.5 (na)
Xu 2020	MSIS 2013	360	153	207	55	305	0	3.6	68.6 (na)	86 (na)	78.4 (na)	78.8 (na)
Xu 2022	MSIS 2013	543	245	298	na	na	0	3.7	69.6 (na)	86.5 (na)	80.9 (na)	77.6 (na)
Yang 2021	ICM 2018	156	57	99	50	106	0	4.2	86 (na)	90 (na)	83.1 (na)	90.8 (na)

Table S5. Characteristics of all included studies evaluating serum fibrinogen.

PJI: periprosthetic joint infection, AF: aseptic failure, CI: confidence interval.

Study (Year)	PJI reference standard	N° Patients	N° PJIs	N° AF	N° knees	N° hips	N° shoulders	Cutoff (pg/mL)	Sensitivity (95%CI)	Specificity (95%CI)	PPV (95%CI)	NPV (95%CI)
Abou El-Khier 2013	Clinical criteria / Other	40	11	29	14	26	0	10.4	100 (na)	90.9 (na)	79 (na)	100 (na)
Ackmann 2020	ICM 2018	119	52	67	71	48	0	10.0	76 (na)	92 (na)	88 (na)	83 (na)
Bottner 2007	Clinical criteria / Other	78	21	57	28	50	0	12.0	95 (na)	87 (na)	74 (na)	98 (na)
Buttaro 2010	Clinical criteria / Other	69	11	58	0	69	0	10.0	36 (30-69)	94 (88-100)	57 (13-100)	88 (na)
Di Cesare 2005	Clinical criteria / Other	58	17	41	na	na	na	10.0	100 (na)	95 (na)	89 (na)	100 (na)
Elgeidi 2014	Clinical criteria / Other	40	11	29	14	26	0	10.5	100 (na)	90.9 (na)	79 (na)	100 (na)
Erdemli 2018	MSIS 2013	88	36	52	59	29	0	16.2	75 (57.8-87.9)	78.85 (65.3-88.9)	71.1 (na)	82 (na)
Ettinger 2015	MSIS 2011	98	41	57	39	57	2	5.12	80 (na)	87.7 (na)	69.6 (na)	92.6 (na)
Gallo 2018	MSIS	240	93	147	116	124	0	12.5	86.7 (77.5-93.2)	89.5 (82.3-94.4)	85.7 (77.7-91.2)	90.3 (84.2-94.2)
Gollwitzer 2013	Clinical / other	35	15	20	15	20	na	1.89	46.7 (na)	95 (na)	87.5 (na)	70.4 (na)
Majors 2019	ICM 2018	59	32	27	59	0	0	9.14	81 (54-95)	63 (43-80)	57 (35-76)	85 (61-96)
Qin 2020 BJR	MSIS 2013	93	37	56	44	48	0	6.7	97.3 (85.8-99.9)	76.79 (63.6-87)	73.5 (na)	97.7 (na)
Villacis 2014	Clinical criteria / Other	34	14	20	0	0	34	10	14 (na)	95 (na)	67 (na)	61 (na)
Worthington 2010	Clinical criteria / Other	46	16	30	0	46	0	9	81 (na)	77 (na)	65 (na)	50 (na)
Xu 2019	ICM 2014	318	129	189	65	253	0	8.6	72.2 (na)	87.4 (na)	77.4 (na)	84.1 (na)
Xu 2021	MSIS 2013	65	34	31	na	na	0	6	83.3 (na)	69.6 (na)	74.1 (na)	80 (na)
Xu 2022	MSIS 2013	543	245	298	na	na	0	8.6	70.9 (na)	86.5 (na)	81.2 (na)	78.3 (na)
Yin 2021	MSIS 2011	35	15	20	16	19	0	23.1	47 (na)	95 (na)	88 (na)	70 (na)
Yu 2020	MSIS 2013	121	20	101	93	28	0	8.1	80 (na)	76.2 (na)	40 (na)	95.1 (na)
Yu 2021	modified ICM 2014	139	62	77	95	44	0	9.0	80.7 (na)	81.8 (na)	78.1 (na)	84 (na)

Table S6. Characteristics of all included studies evaluating serum interleukin 6 (IL-6).

PJI: periprosthetic joint infection, AF: aseptic failure, CI: confidence interval.

Study (Year)	PJI reference standard	N° Patients	N° PJIs	N° AF	N° knees	N° hips	N° shoulders	Cutoff (mg/L)	Sensitivity (95%CI)	Specificity (95%CI)	PPV (95%CI)	NPV (95%CI)
Ackmann 2020	ICM 2018	119	52	67	71	48	0	2.75	38 (na)	94 (na)	83 (na)	66 (na)
Chen Xi 2021	Clinical outcome / Other	75	25	50	40	35	0	2.72	56 (na)	96 (na)	74.1 (na)	95.5 (na)
Chen Y 2022	MSIS 2013	52	26	26	25	27	0	0.27	76 (na)	60 (na)	65.62 (na)	69.57 (na)
Chisari 2021	ICM 2018	259	78	181	226	33	0	0.97	64.3 (na)	96.6 (na)	90 (na)	84.8 (na)
Dong 2024	MSIS 2013	182	64	118	61	121	0	0.62	57.9 (na)	95.7 (na)	95.7 (na)	57.9 (na)
Fernandez-Sampedro 2022	IDSA 2013	180	39	141	62	118	0	1.17	71.8 (58-86)	75.3 (69-83)	45.9 (33.3-58.4)	90.4 (81.1-99.7)
Fu 2019	MSIS 2011	30	15	15	27	3	0	0.85	66.7 (38.4-88.2)	60 (32.3-83.7)	62.5 (35.4-84.8)	64.3 (35.1-87.2)
Grzelecki 2021	ICM 2018	133	68	65	72	61	0	0.45	75 (na)	73.8 (na)	75 (na)	73.8 (na)
Grzelecki 2021	ICM 2018	133	68	65	72	61	0	0.85	33.8 (na)	95.4 (na)	88.5 (na)	57.9 (na)
Hu 2020	MSIS 2013	77	40	37	14	61	2	0.96	87.5 (na)	89.19 (na)	89.74 (na)	86.84 (na)
Kuo 2022	ICM 2018	76	42	34	41	35	0	0.86	84 (na)	34 (na)	62 (na)	63 (na)
Li 2019	ICM 2014	565	95	470	153	412	0	1.25	64.5 (na)	65 (na)	27.8 (na)	89.7 (na)
Liu 2022	MSIS 2011	58	38	20	51	7	0	2.13	31.6 (21.8-40.7)	100 (95-100)	100 (95-100)	43.5 (31.1-50.5)
Maimaiti 2021	MSIS 2013	246	125	121	99	147	0	1.24	65.6 (na)	68.6 (na)	68.3 (na)	65.9 (na)
Muñoz-Mahamud 2022	ICM 2018	93	24	69	66	27	0	0.95	91 (na)	64 (na)	48 (na)	96 (na)
Pannu 2020	ICM 2014	111	49	62	69	42	0	0.85	95.9 (na)	32.3 (na)	52.8 (na)	90 (na)
Qin 2020 JoA	MSIS 2013	122	55	67	44	78	0	1.17	92.7 (82.4-98)	74.63 (62.5-84.5)	75 (na)	92.6 (na)
Shahi 2017	ICM 2014	143	57	86	75	68	0	0.85	89 (77-95)	93 (86-96)	84 (na)	96 (na)
Wu 2020	MSIS 2013	116	81	35	48	71	0	0.41	75.8 (na)	67 (na)	42.4 (na)	89.6 (na)
Xu 2019	ICM 2014	318	129	189	65	253	0	1.02	68.3 (na)	50.7 (na)	44.1 (na)	74.2 (na)
Xu 2021	MSIS 2013	65	34	31	na	na	0	0.8	85.7 (na)	47.8 (na)	66.7 (na)	73.3 (na)
Wang 2020	MSIS 2013	157	51	106	34	123	0	1.22	66.7 (na)	85.9 (na)	69.4 (na)	84.3 (na)

Table S7. Characteristics of all included studies evaluating serum D-Dimer.

PJI: periprosthetic joint infection, AF: aseptic failure, CI: confidence interval.
